# Directional pair distribution function for diffraction line profile analysis of atomistic models

**DOI:** 10.1107/S0021889812050601

**Published:** 2013-01-17

**Authors:** Alberto Leonardi, Matteo Leoni, Paolo Scardi

**Affiliations:** a Department of Civil, Environmental and Mechanical Engineering, University of Trento, Via Mesiano 77, Trento (TN), I-38123, Italy

**Keywords:** directional pair distribution function, line profile analysis, Warren–Averbach analysis, distortion fields, nano-polycrystalline microstructure, molecular dynamics

## Abstract

The concept of the directional pair distribution function is proposed to describe line broadening effects in powder patterns calculated from atomistic models of nano-polycrystalline microstructures. The approach provides at the same time a description of the size effect for domains of any shape and a detailed explanation of the strain effect caused by the local atomic displacement. The latter is discussed in terms of different strain types, also accounting for strain field anisotropy and grain boundary effects. The results can in addition be directly read in terms of traditional line profile analysis, such as that based on the Warren–Averbach method.

## Abstract 2

The concept of the directional pair distribution function is proposed for an atomistic level interpretation of the line profile broadening in powder diffraction patterns of nanocrystalline materials.

## 1. Introduction

Considerable efforts in the study of nanomaterials focus on the visualization of strain at the atomic level. Nevertheless, even the most advanced experimental methods based on coherent diffraction are confined to single isolated nanoparticles and relatively weak strain fields (Thomas, 2008 ▶; Robinson & Harder, 2009 ▶; Watari *et al.*, 2011 ▶). Strains in nano-polycrystalline microstructures are still a challenge to direct visualization techniques, because of the variety of strain sources and the level and complexity of their distribution. In this context, diffraction line profile analysis (LPA), although indirect and much less detailed than coherent diffraction and other microscopies, is still one of the most convenient and widely used experimental techniques. Further insights into the mechanisms and distributions of strains can be produced by atomistic models, in particular by molecular dynamics (MD) simulations (Van Swygenhoven *et al.*, 2000 ▶; Van Swygenhoven, 2002 ▶; Derlet *et al.*, 2005 ▶; Derlet & Van Swygenhoven, 2006 ▶; Cao *et al.*, 2008 ▶; Stukowski *et al.*, 2009 ▶). So far, however, the connection with diffraction LPA has been discussed in terms of simplified integral breadth methods only (Derlet *et al.*, 2005 ▶; Leonardi *et al.*, 2011 ▶): the potential of joining MD simulations with state of the art LPA is still largely unexplored.

In the past decade LPA evolved in the direction of Fourier methods based on physical models of the microstructure (Mittemeijer & Scardi, 2004 ▶). Despite success in many applications, even the most advanced methods of whole powder pattern modelling (Scardi & Leoni, 2002 ▶, 2001 ▶) still rely on a simplified description of lattice defects, and cannot be proven to be univocal in the identification and quantification of the different effects contributing to the line profiles. For example, the uniqueness of the anisotropic line broadening due to the strain field of dislocations, the effects of grain-to-grain deformation fields and the role of grain boundaries are currently open questions. It is not clear to what extent these different sources of lattice deformation can be identified by even the most advanced LPA methods.

MD simulations of nano-polycrystalline microstructures can give useful insights into these problems, provided that the usual concept of strain is reconsidered and related in a more direct way to diffraction LPA. Inhomogeneous strains have a peculiar effect on diffraction, which is sensitive to the distribution of the strain component projected along the scattering vector direction; this strain component needs then to be considered as a function of the separation distance between all possible pairs of atoms (or unit cells, on a coarser size scale) in each given crystalline domain (Warren, 1990 ▶).

As shown by several MD studies, the Debye scattering equation (DSE) is the most direct way to simulate the powder diffraction pattern from a given atomistic model of nano-polycrystalline microstructure (Cervellino *et al.*, 2003 ▶, 2010 ▶; Derlet *et al.*, 2005 ▶; Gelisio *et al.*, 2010 ▶, 2011 ▶), as it just requires atomic positions (strictly speaking, distances between all possible pairs of atoms), with no assumption on the crystal structure or on the presence of lattice defects. While the DSE is rigorous and appealing for simulating the powder pattern, it is not so useful for analysing the effects of strain, separating different sources of line broadening and studying line anisotropy, which makes line broadening different for profiles with different Miller indices.

In the present work we introduce the concept of the directional pair distribution function (D-PDF) to represent the finite size and shape of coherently scattering domains (crystallites) and the local atomic displacement in a way directly legible in terms of diffraction effects. Starting from an atomistic simulation of single crystals as well as of nano-polycrystalline microstructures, the D-PDF allows a separation of all contributing *hkl* line profiles in the powder diffraction pattern. Each line profile can be described in terms of size and strain contributions, in a quite similar way to traditional LPA based on Fourier analysis [*e.g.* the Warren–Averbach (WA) method; Warren & Averbach, 1950, 1952 ▶; Warren, 1990 ▶]. The proposed approach has at least two valuable applications: (i) to validate different LPA methods and better understand their results; (ii) to study MD simulations and their relation with real microstructures in terms of a well known, easy to perform experimental technique like powder diffraction.

The chosen case of study concerns a metallic nano-polycrystalline system made of randomly oriented grains with irregular (though not far from equiaxed) shapes. The system was equilibrated (energy minimized and thermalized) by conventional MD based on the embedded atom method (EAM; Daw & Baskes, 1983 ▶), so that no lattice defects other than the grain boundaries are present. Under these conditions, intergranular strains due to the equilibration process should be the only microstructural effects (besides shape and finite size of the grains) affecting the line profiles. Detailed information on the direction-dependent strain field and its effect on the simulated powder diffraction pattern can be obtained by means of the D-PDF concept. The role of the grain boundaries is also discussed.

## 2. Copper nano-polycrystalline microstructure: generation and strain distribution

**Figure 1:**
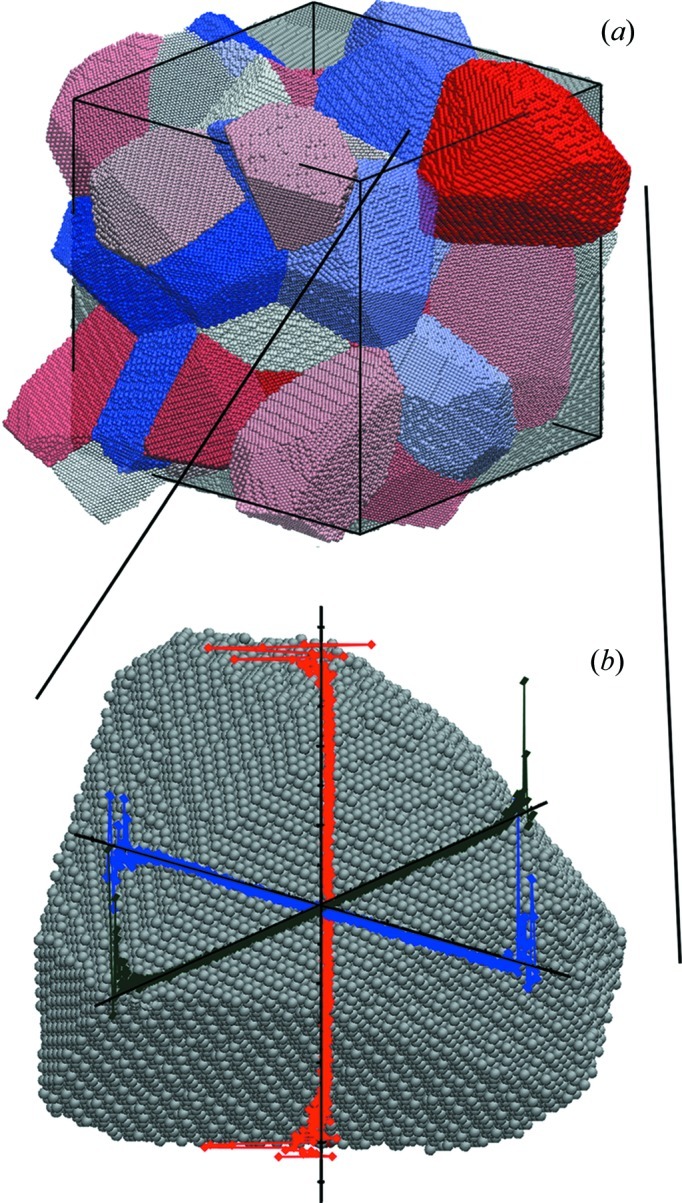
(*a*) Copper nano-polycrystalline microstructure after reconstruction of grains cut across by the PBCs. (*b*) Example of volumetric strain along the 〈*h*00〉 directions in a grain.

A cubic box (side length 260.28 Å) was divided into 50 cells by the recently developed constrained modified Voronoi tessellation (CMVT) (Gross & Li, 2002 ▶; Xu & Li, 2009 ▶; Suzudo & Kaburaki, 2009 ▶; Leonardi, Scardi & Leoni, 2012 ▶; Leonardi *et al.*, 2013*a* ▶). The CMVT allows control of different properties during the generation of the cells. In the present case, the CMVT parameters were set to produce high sphericity (arithmetic mean Ψ = 0.800122) and a lognormal distribution of grain sizes (for the distribution of diameters of equivalent volume spheres: arithmetic mean 82.6 Å, standard deviation 21.4 Å); grains were then filled with about 1.5 million Cu atoms, using a unit-cell parameter of 3.615 Å (Fig. 1 ▶).

**Figure 2:**
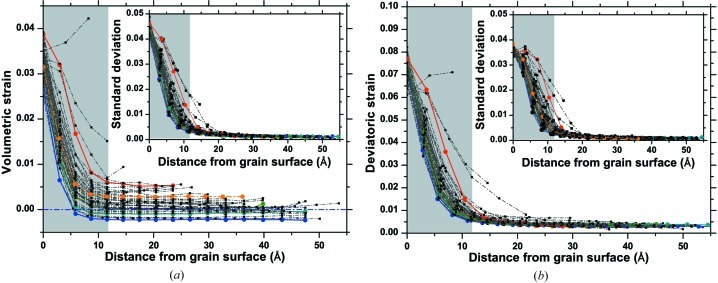
Average volumetric (*a*) and deviatoric (*b*) strain as a function of the position from the grain boundary for the different grains in the cluster. The insets show the variance of the strain distribution.

Equilibration of the starting (‘crystallographic’) system was done by EAM, using the Cu potential reported by Foiles *et al.* (1986 ▶). After the energy minimization, an isothermal–isobaric time integration at 100 K was performed by the *LAMMPS* software (Plimpton, 1995 ▶), reaching equilibrium conditions. The latter were assessed by comparing the deformation field after 1.2 and 2.4 ns of equilibration times, in terms of both volumetric (isotropic) and deviatoric (anisotropic) strain components (Fig. 2 ▶). The zero strain is referred to an equivalent unit cell of 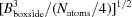, where 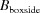 is the equilibrated box side length.

While the MD simulation was performed on a box with periodic boundary conditions (PBCs), grains cut across by the cube faces were joined, so as to create a more plausible system (Fig. 1 ▶ *a*) for the simulation of the powder diffraction pattern by the DSE.

As shown schematically in Fig. 1 ▶(*b*), strain is not constant across the grains: a steep gradient is observed in the outer layers, extending from the grain boundary region inward for about 10 Å. This trend, qualitatively the same for all grains in the cluster, is shown in Fig. 2 ▶: here the mean volumetric (*a*) and mean deviatoric (*b*) strain in each grain are plotted against the distance from the grain boundary. Insets show that the variance of the strain distribution also steeply decreases in the sub-surface region.

**Figure 3:**
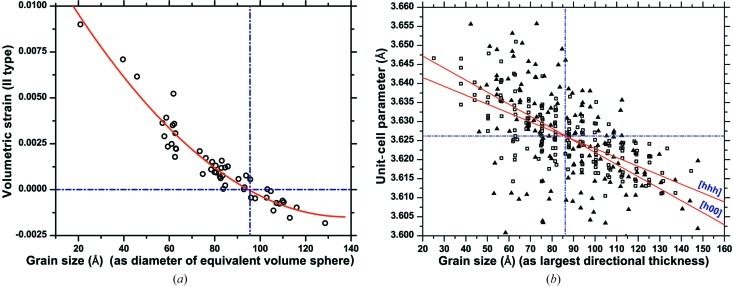
(*a*) Type II volumetric strain as a function of the diameter of the equivalent volume sphere; (*b*) unit-cell parameter as a function of the greatest thickness along the direction considered. See text for details. Dot–dashed lines represent the average values.

While the deviatoric strain decreases to a small constant value in the core region, the value reached by the volumetric component inside grains depends on the grain size. This feature is visible in Fig. 2 ▶(*a*) but it can be seen more directly in Fig. 3 ▶, where the mean unit-cell parameter of each grain is plotted as a function of the diameter of the equivalent volume sphere. Larger grains put in tension the smaller ones, with an apparently nonlinear trend. In Fig. 3 ▶(*b*) it is also possible to see the anisotropy of this deformation, which is higher along [*h*00] than along [*hhh*], respectively corresponding to the elastically softer and stiffer directions in copper.

## 3. Directional pair distribution function (D-PDF)

**Figure 4:**
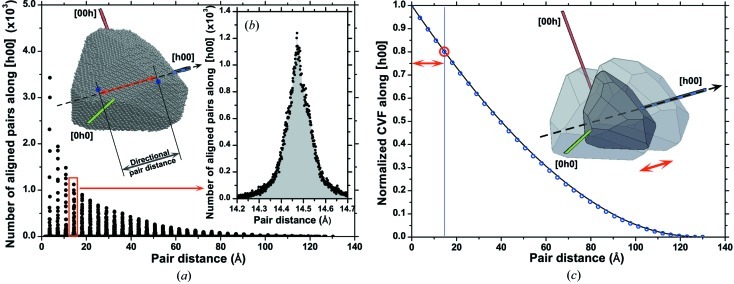
(*a*) Directional pair distribution function for grain No. 33 in the cluster along the [*h*00] direction; (*b*) example of distribution for *L* = 14.485 Å; the area under the curve (shaded) corresponds to the circled value of the normalized common volume function (CVF) in (*c*). (*c*) The trend of the normalized area of 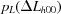 as a function of the pair distance. The inset in (*c*) illustrates the concept of the ‘ghost’ (see text for details).

The D-PDF is a convenient concept to represent strain effects on the diffraction line profiles. It is obtained by counting the number of atom pairs for each distance *L* along the [*hkl*] direction of the scattering vector in a given grain of the cluster. As shown in Fig. 4 ▶, the projection of the atom–atom dis­place­ment Δ*L* along [*hkl*] is actually considered: the D-PDF is then made of a series of histogram distributions, centred on the mean distance between *n*th neighbour atoms, ranging from the closest neighbour to the longest distance in the grain along [*hkl*]. Each distribution 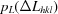 can be calculated as described above, along [*hkl*] of a given grain, or be averaged over all crystallographically equivalent 〈*hkl*〉 directions in the grain, or be averaged over all equivalent directions of all grains in the cluster (so-called super D-PDF).

It is important to note that the D-PDFs along symmetrically equivalent directions are in general different, as the strain field caused by the neighbouring grains is not subject to any symmetry restrictions. These differences, however, tend to disappear in the average for a given grain or for the entire cluster.

As an example, Fig. 4 ▶(*a*) shows the D-PDF along the [*h*00] direction of grain number 33 in the cluster, with one such distribution shown in detail in Fig. 4 ▶(*b*). The trend of the normalized area of 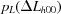 as a function of the pair distance in shown in Fig. 4 ▶(*c*). This quantity is equivalent to the common volume function (CVF), as introduced by Stokes & Wilson (1944 ▶), representing the intersection volume between a crystalline domain and its ‘ghost’, *i.e.* the same domain shifted a distance *L* along [*hkl*] (inset of Fig. 4 ▶ *c*). As shown by several authors (Wilson, 1962 ▶; Guinier, 1963 ▶; Warren, 1990 ▶), within reasonable approximations the Fourier transform (FT) of the CVF gives the so-called size component of the diffraction line profile, because of the finite size of the crystalline domain along the given [*hkl*] direction. As the D-PDF is not normalized, the corresponding diffraction line profile obtained by FT includes the appropriate weight (volume or number of atoms) for the given grain.

The D-PDF concept allows for a simple and reliable way to separate the effects of domain size/shape from those due to lattice defects and microstructural features in general. While the 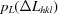 area is related to the domain size/shape component of the line profile, the width and shape of the D-PDF provide a detailed description of the atomic dis­place­ment over different distances. As shown below, this is the same representation of strain effects on line profiles as that used by traditional LPA based on Fourier analysis, like the WA method (Warren & Averbach, 1950 ▶, 1952 ▶; Warren, 1955 ▶; Warren, 1959 ▶) and methods proposed by Stokes and Wilson (Stokes & Wilson, 1944 ▶; Eastabrook & Wilson, 1952 ▶).

It is convenient to introduce the strain over a distance *L* along the [*hkl*] direction, 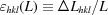, and the scattering vector modulus, 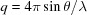, where θ is half the scattering angle and λ is the wavelength of the incident radiation. As originally shown by Stokes and Wilson (Stokes & Wilson, 1944 ▶; Wilson, 1962 ▶), the FT of the peak profile component due to the local atomic strain, 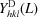, can be reasonably approximated as
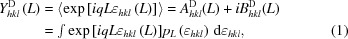
where the 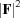 term (related to the structure factor, *F*) is omitted for simplicity. As shown in equation (1), 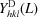 is the Fourier transform of the D-PDF, which is a complex quantity unless the D-PDF is symmetrical. In equation (1) the D-PDF was written for strain, which is straightforward considering that 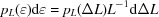.

The normalization used in equation (1) is such that 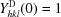, so it is convenient to represent the CVF in normalized form too:
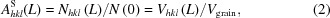
where 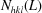 is the number of atom pairs at distance *L* along [*hkl*] and 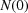 is the total number of atoms in a given grain; correspondingly, 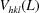 is the CVF along [*hkl*] and 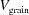 the volume of the given grain. It is worth noting here that the present description in terms of D-PDF is on a finer and generally more precise atomic scale, whereas traditional LPA usually considers unit cells as smallest units (Wilson, 1962 ▶; Warren, 1990 ▶).

Within the limit of Stokes & Wilson’s theory (Stokes & Wilson, 1944 ▶; Eastabrook & Wilson, 1952 ▶), the FT of the overall line profile for the [*hkl*] direction in a given grain of the cluster is
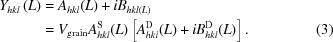
The distribution 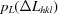 is frequently assumed to be symmetrical (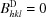) and in particular Gaussian, so that
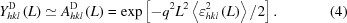
These are the underlying hypotheses of the WA method (Warren & Averbach, 1950 ▶; Warren & Averbach, 1952 ▶; Warren, 1955 ▶, 1959 ▶, 1990 ▶), which can be shown to be still approximately valid even if the distribution is not exactly Gaussian, provided it is a bell-shaped symmetrical distribution function (Warren, 1959 ▶). As further discussed below, the D-PDFs for the studied cluster are neither Gaussian nor symmetrical.

The powder diffraction pattern for a cluster of grains can be obtained by adding up all contributions as
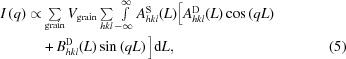
where the proportionality factor includes 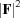, several constants and known trigonometric terms, whereas the actual integration limits are determined by the longest atom–atom distance along [*hkl*] in the given grain. The two sums extend, respectively, to all grains and to all crystallographically equivalent directions.

**Figure 5:**
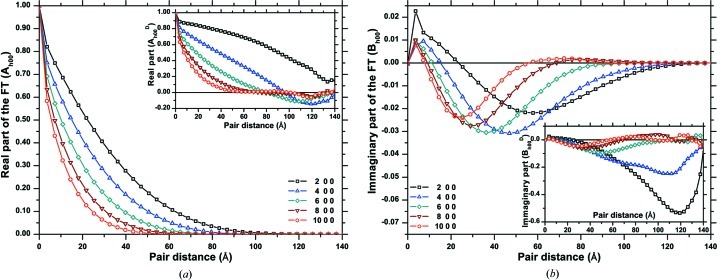
Real (*a*) and imaginary (*b*) parts of the Fourier transform of the peak profile; the example refers to the {*h*00} family of diffraction peaks (see text for details).

As an example of application of the D-PDF, Figs. 5 ▶(*a*) and 5 ▶(*b*) show, respectively, the real and imaginary parts of the FT for several orders of reflections belonging to the {*h*00} family, 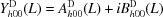. The real part decays increasingly faster for higher orders of reflections, whereas the imaginary part sensibly deviates from zero, as an effect of the asymmetry of the D-PDFs. The trends of 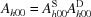 and 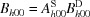 (assuming 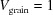 for simplicity) show the combined effect of domain size and strain: the decay of the real part is smooth and demonstrates that the integration limits in equation (5) do not need to extend beyond *ca* 120 Å, in this specific case, as an effect of the finite size of the domain. The fact that different orders of reflections belonging to the same family have different trends, with a faster decay for the higher orders (*i.e.* larger *q* values), clearly demonstrates the presence of a strain broadening component.

## 4. Powder pattern from a nano-polycrystalline microstructure

As already pointed out in the *Introduction* [Sec sec1], the Debye scattering equation (Cervellino *et al.*, 2003 ▶, 2010 ▶; Derlet *et al.*, 2005 ▶) is the most straightforward and correct way to calculate the powder diffraction pattern for an atomistic model. The DSE makes no assumptions on crystalline structure and lattice defects, as it is based only on correlations between all possible pairs of atoms. As such it can be computationally demanding (although nowadays entirely viable; Gelisio *et al.*, 2010 ▶) but quite rigorous, so that we can consider the DSE result as an ‘experimental’ pattern, with which the D-PDF analysis discussed above can be compared.

The goal here is to test the D-PDF approach, to better understand and validate the traditional LPA methods and their results, but also to study the features of a nano-polycrystalline microstructure obtained by MD in terms of a well known and simple experimental technique like powder diffraction.

**Figure 6:**
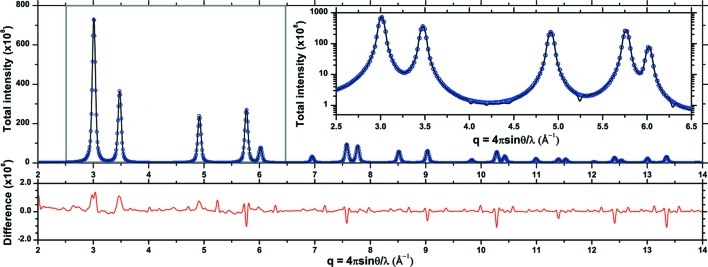
Powder diffraction pattern of the cluster of Fig. 1 ▶ as obtained by the DSE (circles) and the D-PDF approach [equation (5), line]. The differences are shown below. The inset highlights details in the peak tail region.

As a first test we considered the starting cluster, just after the atom filling procedure but before any energy minimization and thermalization steps. This corresponds to a system of perfect crystalline grains, where the only effect on line profiles is that of the finite domain size. As shown in Fig. 6 ▶, the match between the DSE pattern and that generated by using equation (5) is remarkably good (Leonardi *et al.*, 2013*c* ▶; Leonardi, Leoni *et al.*, 2012 ▶). This result is to some extent expected, as no strain and no deviation from a perfect crystalline order is present; however, this step is important to assess the quality of the hypotheses underlying equation (5), such as the tangent plane approximation (Beyerlein *et al.*, 2011 ▶) and the lack of grain–grain correlations, which are intrinsically considered by the DSE.

The MD equilibration procedure introduces strains in the system, leading to a static component of the atomic displacement, which adds to the dynamical component due to thermal vibrations. At any given instant (frame) of the MD trajectory, the atom–atom displacement can be written as 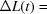 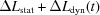. Thermal effects, at least in an approximate way, might be added to the simulation of the powder pattern [*e.g.* by introducing a Debye–Waller factor and temperature diffuse scattering (Warren, 1990 ▶; Beyerlein *et al.*, 2012 ▶)]. However, for the purpose of studying the static component it is more convenient to introduce the concept of time-averaged atomic coordinates (TACs): the atomic coordinates of all atoms in the studied system are averaged over a suitably large number of time frames of the MD trajectory, so that 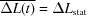. In this way we can get rid of the dynamical component of atomic displacement and refer the diffraction LPA to the static component only. Once the TACs are known, the DSE pattern is easily obtained as explained before. The D-PDF pattern is then built according to equations (1) and (5).

**Figure 7:**
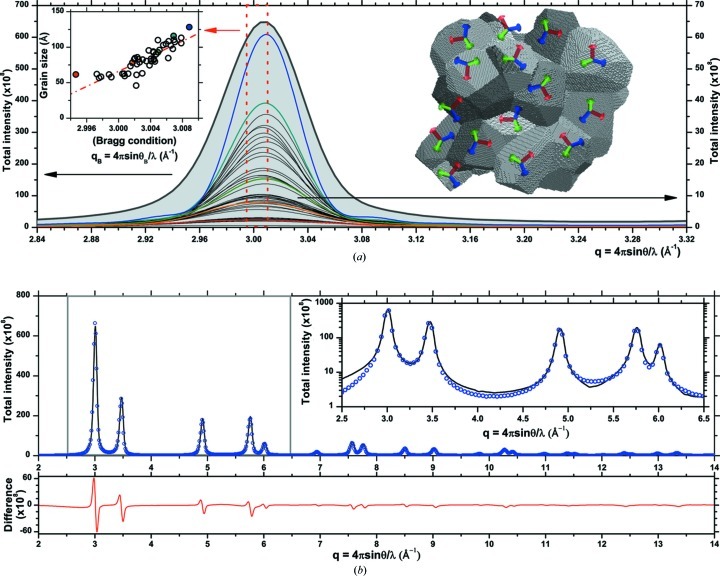
Powder patterns of the system of Fig. 1 ▶ after MD equilibration. (*a*) Detail of the 200 peak as built from the D-PDF method of equation (5) [whole microstructure (thickest line), single direction of each grain (thin lines)]; (*b*) comparison between DSE (circles) and D-PDF patterns (line), with difference plot below.

If we now move to the system after MD equilibration, Fig. 7 ▶(*a*) shows the detail for the 200 peak, as obtained by adding all {200} reflections in the cluster. While the right inset highlights the trend for the 〈*h*00〉 directions in different grains, the plot in the left inset shows an interesting feature of the position (*q*_B_) of the *h*00 peaks in the cluster, which changes as a function of the size of the corresponding grain, as an effect of the strain dependence on the grain size (*cf.* Fig. 3 ▶).

Fig. 7 ▶(*b*) shows a comparison between patterns for the equilibrated system, as obtained by DSE and D-PDF. Small but visible differences are expected and suggest interesting features of the strain field. While the DSE includes all regions of the cluster independently of their order (whether they are crystalline or amorphous), the D-PDF refers by definition to an underlying crystalline lattice: local atomic displacement is allowed (D-PDF position, width and shape) but is in any case bounded by the average crystalline framework. The residual in Fig. 7 ▶(*b*) is therefore related to a highly strained region, somewhere in between a highly distorted crystalline lattice and an amorphous phase, which is very likely the grain boundary area. Moreover, while the DSE is sensitive to all possible correlations within the cluster, *i.e.* between atoms inside each grain as well as between atoms of different grains (Leonardi *et al.*, 2013*b* ▶), the D-PDF considers just those between atoms inside grains.

**Figure 8:**
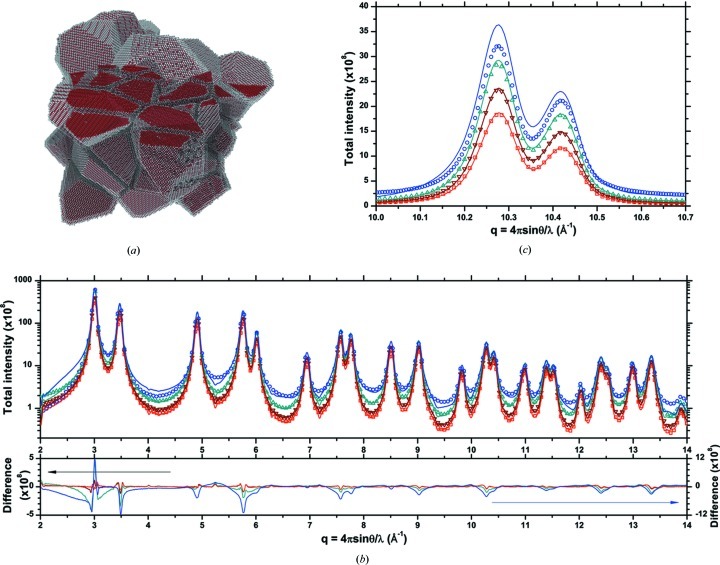
(*a*) MD-equilibrated cluster of Fig. 1 ▶ after removal of one atomic layer from each grain. (*b*) DSE pattern for the equilibrated cluster (open circles) and after removal of one (upward triangles), two (downward triangles) and three (squares) layers. The line refers to the corresponding patterns calculated from the D-PDF approach of equation (5). The difference between the DSE and D-PDF patterns is shown below. (*c*) A detail for the 531 and 442/600 peaks.

To test this hypothesis, the DSE and the D-PDF patterns were calculated again on the same equilibrated cluster but after removing, respectively, one, two or three external atomic layers from all grains. In this way the grain boundary region is progressively eliminated (Fig. 8 ▶).

As shown in Fig. 8 ▶(*c*) for a restricted angular region at relatively high *q*, the match between the DSE and the D-PDF patterns is markedly improved by removing surface layers. As soon as the first layer is removed, just fully coordinated atoms are considered and most of the discrepancy disappears (Fig. 8 ▶ *b*). Besides zeroing the peaks in the low-*q* region of the residual, this step eliminates nearly completely the diffuse scattering at higher *q* values. This last detail is visible by comparing the inset of Fig. 7(*b*) and Fig. 8 ▶(*b*).

A progressive removal of layers can thus help the investigation of the degree of disorder in the grain boundary region, the contribution of this region to the strain field in the grains, and the corresponding line profile broadening and diffuse scattering. The results suggest that the grain boundary region, as obtained from the adopted MD equilibration procedure, is highly distorted: it is probably not truly crystalline, but it is not even a completely amorphous phase (Leonardi *et al.*, 2013*b* ▶). Effects on line profiles are clearly visible, even more so the smaller the crystalline domains.

## 5. D-PDF and r.m.s. strain

**Figure 9:**
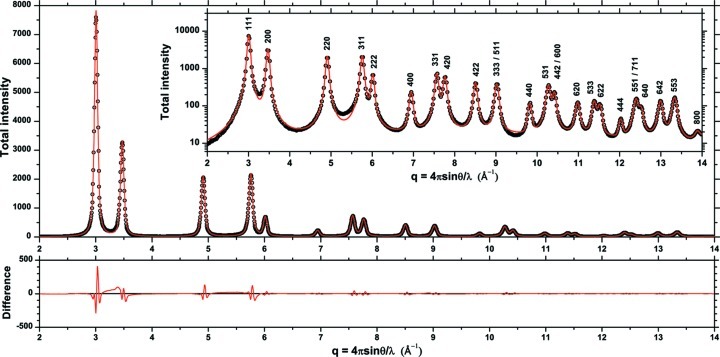
Result of profile fitting of the pattern given by the Debye scattering equation for the studied metallic cluster: DSE data (circles), best fit with pseudo-Voigt functions (line) and difference between the two (residual, line below). Corresponding Miller indices are shown in the log-scale plot in the inset.

**Figure 10:**
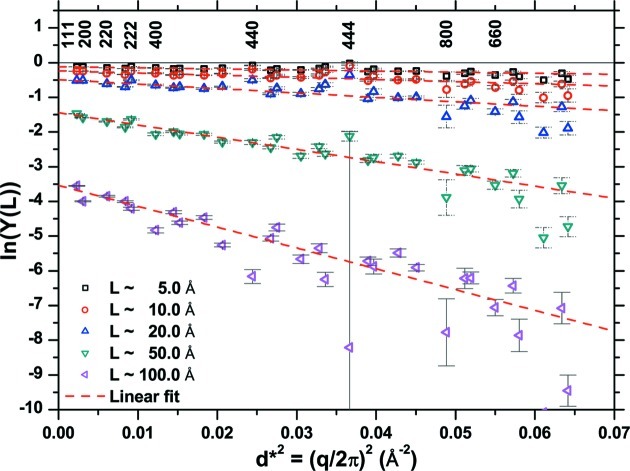
Warren–Averbach plot: logarithm of the Fourier transform of the peak profiles (from profile fitting in Fig. 9 ▶) as a function of the square of the scattering vector (*q*/2π), for a selection of pair distances *L* (5, 10, 20, 50, 100 Å). Points with the same (*q*/2π) correspond to a given set of Miller indices, shown on top for the {*hhh*} and {*h*00} families (333 and 600 are not considered as they overlap with other reflections).

It is interesting to consider again the DSE pattern as an ‘experimental’ pattern from a nano-polycrystalline system, and make a traditional line profile analysis. As a first step, which is ordinary practice in real cases of study, profile fitting is used to separate contributions from the different, strongly overlapping peaks (Dong & Scardi, 2000 ▶). The fitting results shown in Fig. 9 ▶ are reasonably good, although less satisfactory for the peaks at lower *q*, which are more affected by the grain boundary and the grain–grain correlations discussed in the previous section. From this analysis (Dong & Scardi, 2000 ▶) it is straightforward to obtain the plot of Fig. 10 ▶, *i.e.* the logarithm of the Fourier transform of the line profiles as a function of 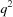 [actually 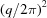 for historical reasons] for different pair distances *L*.

According to Warren and Averbach, the observed data can be described as (Warren & Averbach, 1950 ▶, 1952 ▶; Warren, 1955 ▶, 1959 ▶, 1990 ▶)
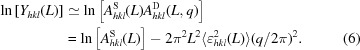
Information on the domain size and variance of the strain distribution 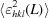 [or of the displacement distribution, 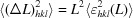] can be obtained, respectively, from the intercept and slope of the trends in Fig. 10 ▶ considered for different pair distance values. To properly account for a possible dependence on the crystallographic direction, the procedure is performed separately for peak profiles belonging to different {*hkl*} families (Warren, 1959 ▶), although an analysis involving all observed peaks can also be informative.

**Figure 11:**
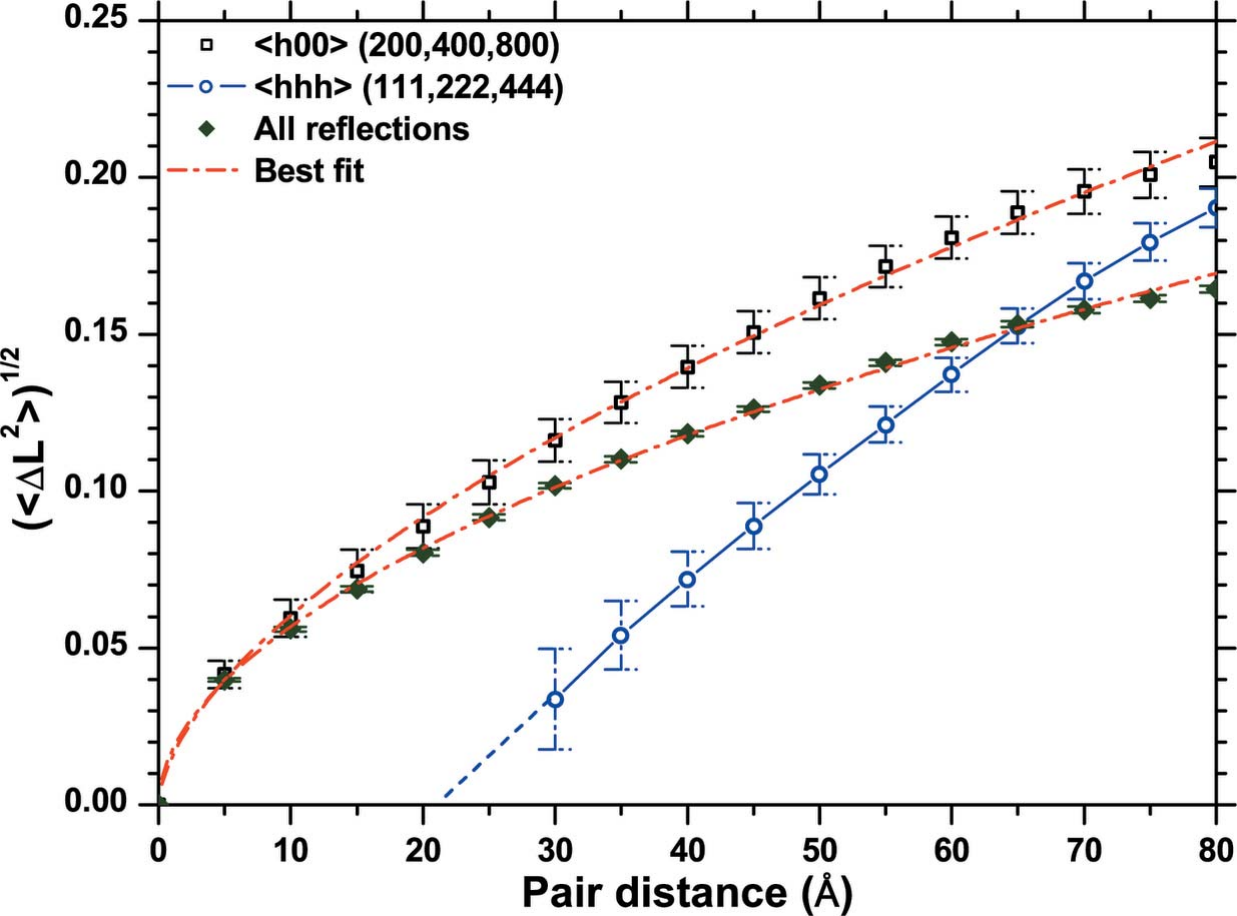
Warren plot: standard deviation of the distribution as a function of the atomic pair distance along 〈*h*00〉 (squares) and 〈*hhh*〉 (circles), and the average over all 〈*hkl*〉 (filled diamonds); the best fit (dash–dot line) refers to a power law (see text for details).

Fig. 11 ▶ shows the standard deviation 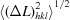 as a function of *L* for 111/222/444 and 200/400/800 (333 and 600 were excluded as they overlap, respectively, with 511 and 442). As shown in the original paper by Warren & Averbach (1950 ▶), all trends should start from the origin: while this happens for the {*h*00} family, it is not the case for the {*hhh*} family, which gives a trend crossing the abscissa just above *L* = 20 Å. This is an artefact at least partly caused by systematic errors in profile fitting (*e.g.* of the 111 peak, see Fig. 9 ▶) and by the severe overlapping of the peak profiles (especially relevant for the weak 444 peak), which are typical problems also in real cases of study. However, as expected for the elastic anisotropy of copper, the r.m.s. displacement is higher for {*h*00} than for {*hhh*}, while results from other {*hkl*} families fall in between these two limits.

Also shown in Fig. 11 ▶ is the result from all observed peak profiles: this procedure disregards the elastic anisotropy but helps in averaging the effects of a non-perfect fitting of the peak profiles.

Several studies propose an interpretation of the trends in Fig. 11 ▶. According to Adler & Houska (1979 ▶), data of Fig. 11 ▶ should obey a simple power law, 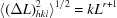, with 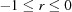. The value found for {*h*00} is *r* = −0.44 (2), whereas the fit to the data from all peak profiles gives *r* = −0.47 (1). A value around *r* = −0.5 is considered as typical of cold-worked metals, and would be a result of the non-uniform strain field of dislocations (Adler & Houska, 1979 ▶).

**Figure 12:**
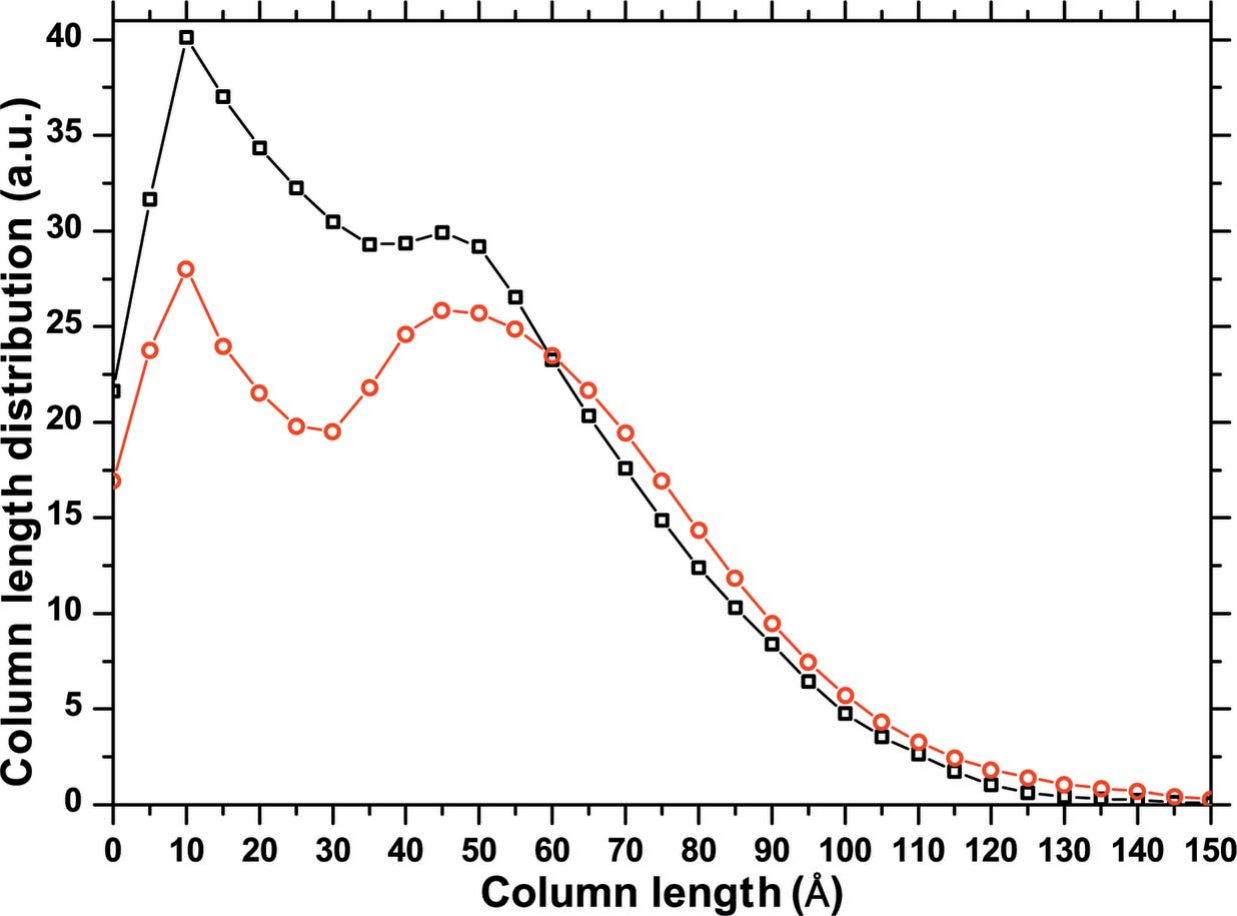
Column length distributions along 〈*h*00〉, as obtained from the Warren–Averbach analysis of profile data from the DSE pattern (circles) and directly from the D-PDF analysis (squares).

Before considering the correct trend of 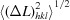 from the D-PDF analysis, it is worth analysing the results on the domain size obtained from the intercepts in Fig. 10 ▶. For simplicity we consider the {*h*00} family, which gave a plausible trend in Fig. 11 ▶. It can be shown that, in a plot of the size coefficients 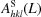 as a function of *L*, the intercept with the abscissa of the tangent to the curve at *L* = 0 is a surface-weighted mean domain size, 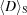; the second derivative of 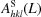, instead, is proportional to the length distribution of the domain along the scattering vector direction (the so-called column length distribution), from which a volume-weighted mean domain size 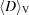 can be calculated (Bertaut, 1950 ▶, 1952 ▶; Warren, 1990 ▶). Fig. 12 ▶ shows the column length distribution along 〈*h*00〉; the mean sizes are 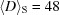
 Å and 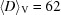
 Å.

These results can be compared with the values provided by the D-PDF analysis, which can be considered ‘exact’ in that they are directly obtained from the known parameters of the cluster. The CVF is obtained from the area below the D-PDF curves (*e.g.* those in Fig. 4 ▶), and after normalization to the grain volume it provides the size coefficients for the given grain and [*hkl*] direction. 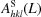 is then calculated by averaging the coefficients over all grains in the cluster, and the second derivative provides the column length distribution. This procedure, applied to the 〈*h*00〉 directions, gives 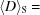 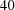
 Å and 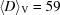
 Å, and the column length distribution shown in Fig. 12 ▶. Despite the quite different procedures involved, the column length distributions are remarkably similar. The profile fitting/Warren–Averbach method tends to overestimate the size, the discrepancy being larger for 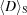 than for 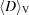, as the former is more influenced by the shorter lengths; these in turn depend more directly on the peak tails, which are less accurately described by the profile fitting.

**Figure 13:**
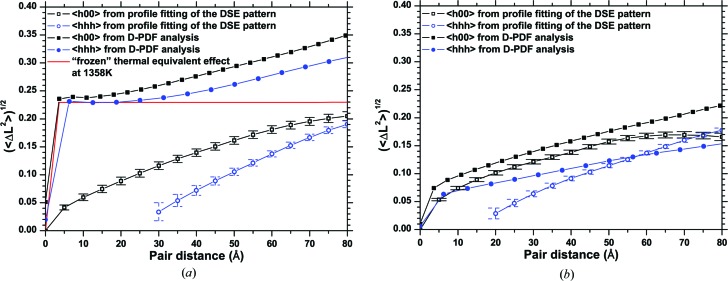
Warren plot comparing results of Fig. 11 ▶, obtained from the Warren–Averbach analysis of profile data from the DSE pattern (filled symbols), with the standard deviation of the strain distribution directly calculated from the D-PDFs. Results refer to the microstructure of Fig. 1 ▶ after MD equilibration (*a*) and after removal of one atomic layer from the surface of all grains in the cluster (*b*). The full line in (*a*) indicates the equivalent thermal strain at melting temperature.

Standard deviations of the strain distribution are easily calculated numerically for each D-PDF curve, and averaging this result over equivalent directions of all grains provides 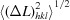 for the cluster. The trends for the 〈*h*00〉 and 〈*hhh*〉 directions are shown in Fig. 13 ▶(*a*), together with the corresponding results from the Warren–Averbach analysis.

**Figure 14:**
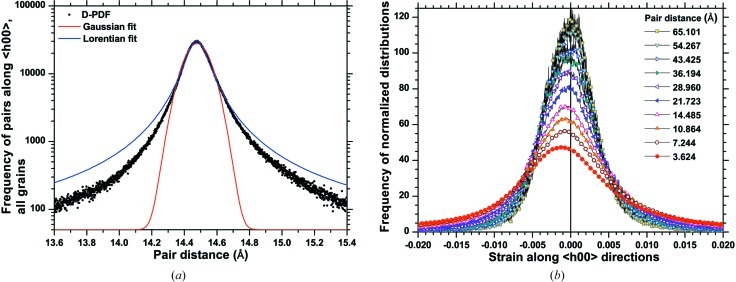
(*a*) Example of a pair distance distribution (average over the whole cluster for the 〈*h*00〉 directions) with the best fits of a Gaussian and of a Lorentzian function; (*b*) corresponding strain distributions for different *L* values.

While the agreement between results on domain size is good, the standard deviations of the displacement distribution look completely different. Reasons for this discrepancy can be found in the fine features of the D-PDF curves, and in the simplifying assumptions underlying the Warren–Averbach method. As shown in Fig. 14 ▶, the assumption of a Gaussian and symmetrical 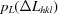 is far from being correct; moreover, the shape of the strain distribution changes for different pair distances (Fig. 14 ▶ *b*).

However, the non-Gaussian (and asymmetrical) nature of 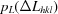 alone cannot explain the large discrepancy in the results of Fig. 13 ▶. An interesting feature, visible in the log-scale plot of Fig. 14 ▶(*a*), is the constant ‘background’, *i.e.* the fact that the distributions do not fall to zero with increasing distance from the expected (perfect crystal) value of atomic pair distance *L*. This constant component is due to the highly disordered grain boundary region, which contributes to the peak profiles with an atomic displacement effect independent of *L*. This is similar to the effect explained by Warren and Averbach for the thermal vibrations, with the important difference that the strain component involved here is static, as the dynamic component was removed before producing the DSE pattern.

**Figure 15:**
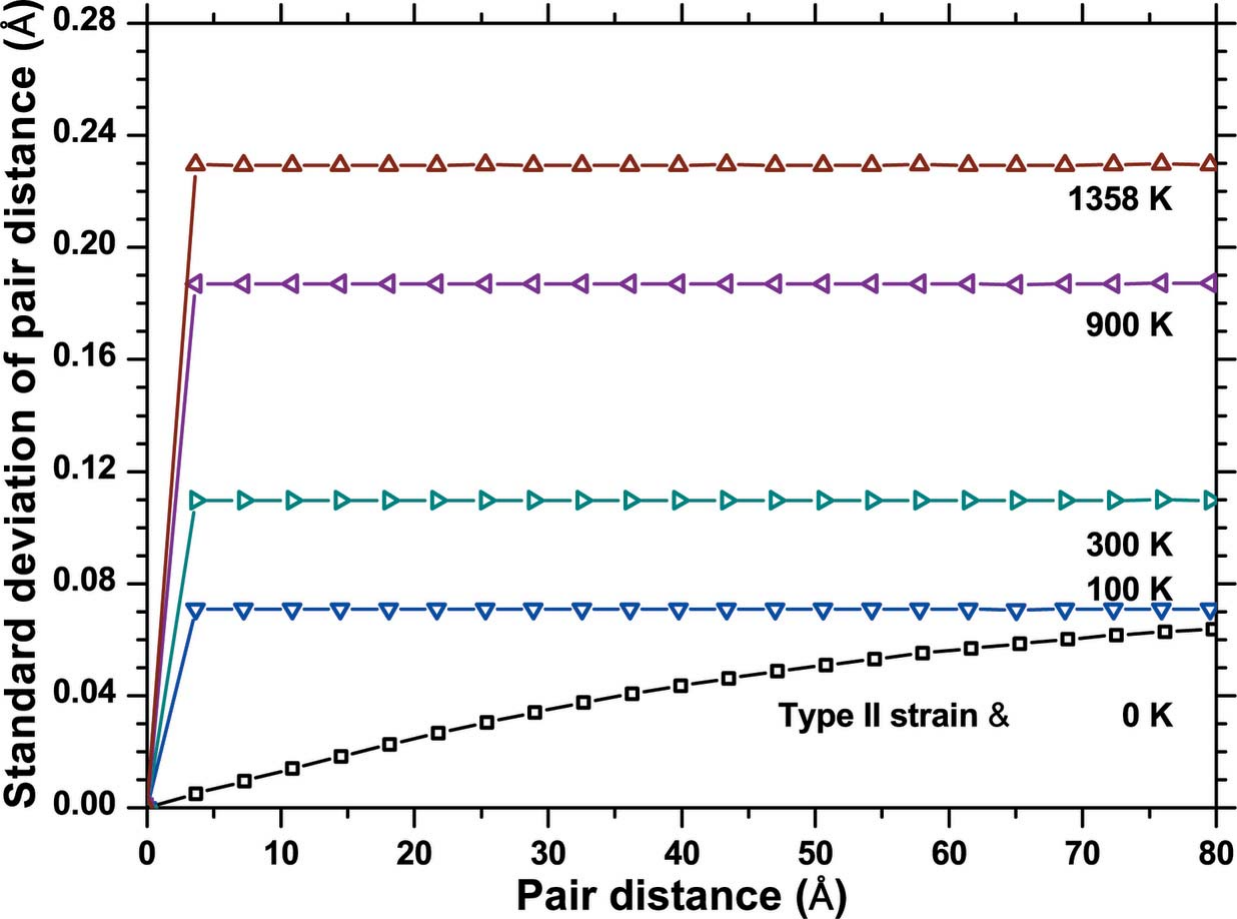
Standard deviation of a strain distribution simulated by adding increasing levels of random static disorder, with indication of the corresponding equivalent temperature. The type II strain effect is also shown (see text for details).

To test this hypothesis, starting from the ‘crystallographic’ system (*i.e.* before any energy minimization and thermalization procedure), we added increasing levels of random static disorder (as in a ‘frozen’ thermal effect). The D-PDF analysis gives the 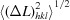 trends in Fig. 15 ▶(*a*), showing a constant increase with the equivalent temperature, up to 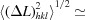 0.23 for an effect equivalent to the melting point of copper (*ca* 1358 K). As shown by the dashed line in Fig. 13 ▶, this value agrees quite well with the sharp step in the 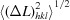 trend.

A further contribution to the observed strain is of course given by the non-uniform strain across grains, and from grain to grain, shown in Figs. 2 ▶ and 3 ▶. These are responsible for the *L*-dependent component of the trend in Fig. 13 ▶, and could be schematically labelled as strains of II (intergranular) and III (intragranular) type (Van Houtte, 1993 ▶; Hutchings *et al.*, 2005 ▶). For a simplified estimate of this complex effect, we added a pure type II strain to the crystallographic cluster. This was done by changing the mean unit-cell parameter of each grain according to the values of Fig. 3 ▶, resulting in a nearly linear increase of the standard deviation with the pair distance (Fig. 15 ▶). A combination of the two effects, type II strain and random static disorder equivalent to 1358 K, explains at least qualitatively the trend observed in Fig. 13 ▶. The differences are due to the simplification of considering a type II strain only, whereas the strain also changes inside each grain with a type III component. Elastic anisotropy should be taken into account too.

**Figure 16:**
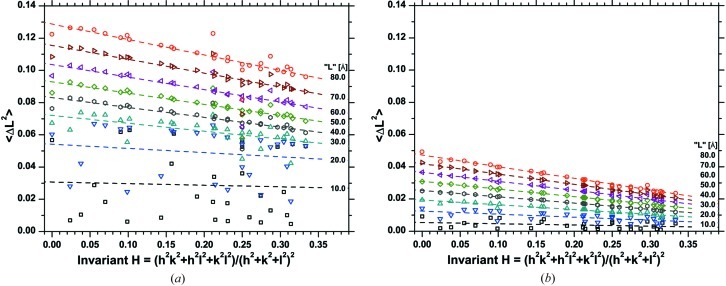
Variance of the strain distribution as a function of the invariant form *H*, for different values of the pair distance, *L*. Results refer to the MD-equilibrated system before (*a*) and after (*b*) removal of one atomic layer from all grains.

Indeed, as shown in Fig. 13 ▶, the standard deviation of the atomic displacement distribution for the two extreme directions 〈*h*00〉 and 〈*hhh*〉 depends on the crystallographic direction. Further evidence is provided by Fig. 3 ▶(*b*), where a directional dependence of the trend of the unit-cell parameter *versus* the largest thickness is shown. This anisotropic behaviour can be investigated following the idea originally proposed by Stokes and Wilson (Sibson, 1980 ▶). In Fig. 16 ▶ the variance 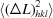 for different *L* values is plotted as a function of the invariant for the (cubic) Laue group of copper, 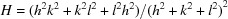. Excluding the very low *L* values (up to about 20 Å), the trend is reasonably linear. It should be considered that the first step in the D-PDF is of different length for the different *hkl*s (high indices can have rather long distances between neighbours). This can explain the scatter observed for low *L* values, where the highly disordered grain boundary contribution can play a major role.

It is also possible to plot the standard deviation 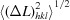 as a function of *H* (Fig. 16 ▶). The fit is again acceptably linear, but the best regression is obtained for the plot of 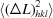 *versus H*. We could further speculate on this result, to find the best functional dependence of 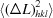 on *H*, but the main conclusion is that this correlation is a consequence of the elastic anisotropy of the metal. A similar effect is also observed when dislocations are responsible for line broadening and can be expressed *via* the contrast factor concept (Wilkens, 1969 ▶, 1970 ▶; Martinez-Garcia *et al.*, 2009 ▶), but in our case, quite evidently, no dislocations are present. This suggests great caution is required in univocally attributing line profile broadening anisotropy to dislocations, which, quite evidently, are just one possible source for this directional effect.

**Figure 17:**
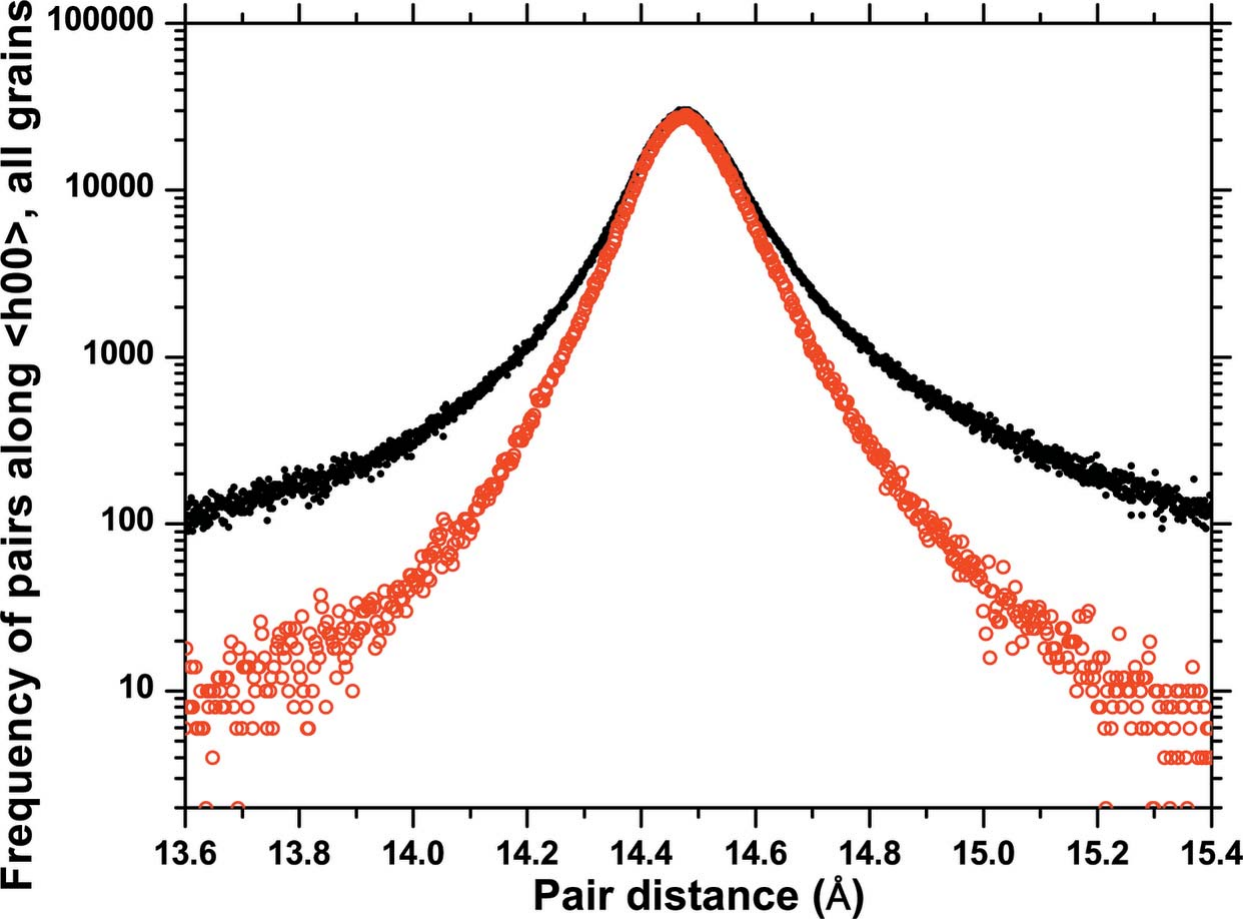
Example of a distribution from Fig. 14 ▶(*a*) for the MD-equilibrated system (filled circles) and after removal of one atomic layer from all grains (open circles).

The analysis presented in this section can be repeated on the DSE patterns of Fig. 8 ▶. After removal of one surface layer from each grain (corresponding to removal of all atoms with coordination lower than 12), all D-PDFs are in general much narrower than those of the cluster completely equilibrated by MD. In addition (see Fig. 17 ▶), the removal of a surface layer drastically reduces the extension of the distribution tails and of the constant component, which was interpreted as a ‘frozen’ thermal-like effect of the disordered grain boundary region. An additional layer removal further extends this effect.

The standard deviation of the atomic displacement is consequently smaller than in the starting cluster. The observed trend in the Warren plot (Fig. 13 ▶ *b*) is much more ‘regular’, *i.e.* as expected from the Warren–Averbach model (Warren & Averbach, 1950 ▶), as a consequence of the much reduced (nearly eliminated) constant contribution of the grain boundary. This further confirms the role of the grain boundary as a region with high disorder but which still contributes to the coherent scattering of the crystalline grains: not all scattering from the grain boundary is therefore diffuse scattering. As a further support to this interpretation, Fig. 13 ▶(*b*) shows that the WA analysis on the DSE pattern after removal of one surface layer from each grain leads to results much closer to those from the D-PDF analysis than for the starting cluster (*cf.* Fig. 13 ▶ *a*). Finally, it is also worth noting that after removal of one layer the anisotropy of the line broadening is more evident, even if the total strain is lower than in the original cluster. As shown in Fig. 16 ▶(*b*), the plot of the variance as a function of *H* gives linear correlations for any *L* value with a lower data scatter than in Fig. 16 ▶(*a*).

## 6. Conclusions

The concept of the directional pair distribution function has been introduced to support a better understanding of line broadening effects in the diffraction peak profiles from nano-polycrystalline microstructures. The new concept, illustrated for a simple system made up of (nearly equiaxed) nanocrystalline Cu grains equilibrated by MD, can easily be applied to any simulated microstructure, including lattice defects and crystalline domains of any shape.

The D-PDF analysis shows in detail how the local atomic displacement influences line profiles, also taking into account the anisotropy of the strain distribution. Most importantly, the D-PDF approach leads to results equivalent to a traditional line profile analysis based on a Fourier formalism, such as the method of Warren and Averbach, thus providing a direct possibility to understand the meaning of LPA results in terms of an atomistic model of the microstructure. This capability can be especially useful for a correct interpretation of experimental LPA results and can provide further insights into the analysis of diffraction phenomena.

## References

[bb1] Adler, T. & Houska, C. R. (1979). *J. Appl. Phys.* **50**, 3282–3287.

[bb2] Bertaut, E. F. (1950). *Acta Cryst.* **3**, 14–18.

[bb3] Bertaut, E. F. (1952). *Acta Cryst.* **5**, 117–121.

[bb4] Beyerlein, K. R., Leoni, M. & Scardi, P. (2012). *Acta Cryst.* A**68**, 382–392. 10.1107/S0108767312009853 22514070

[bb5] Beyerlein, K. R., Snyder, R. L. & Scardi, P. (2011). *J. Appl. Cryst.* **44**, 945–953.

[bb6] Cao, A., Wei, Y. & Ma, E. (2008). *Phys. Rev. B*, **77**, 195429.

[bb7] Cervellino, A., Giannini, C. & Guagliardi, A. (2003). *J. Appl. Cryst.* **36**, 1148–1158.

[bb8] Cervellino, A., Giannini, C. & Guagliardi, A. (2010). *J. Appl. Cryst.* **43**, 1543–1547.

[bb9] Daw, M. S. & Baskes, M. I. (1983). *Phys. Rev. B*, **29**, 6443–6453.

[bb10] Derlet, P., Van Petegem, S. & Van Swygenhoven, H. (2005). *Phys. Rev. B*, **71**, 024114.

[bb11] Derlet, P. M. & Van Swygenhoven, H. (2006). 5th International Conference on Synchrotron Radiation in Materials Science (SRMS-5), 30 July–2 August 2006, Chicago, IL, USA.

[bb12] Dong, Y. H. & Scardi, P. (2000). *J. Appl. Cryst.* **33**, 184–189.

[bb13] Eastabrook, J. N. & Wilson, A. J. C. (1952). *Proc. Phys. Soc. B*, **65**, 67–75.

[bb14] Foiles, S., Baskes, M. & Daw, M. (1986). *Phys. Rev. B*, **33**, 7983–7991. 10.1103/physrevb.33.7983 9938188

[bb15] Gelisio, L., Azanza Ricardo, C. L., Leoni, M. & Scardi, P. (2010). *J. Appl. Cryst.* **43**, 647–653.

[bb16] Gelisio, L., Azanza Ricardo, C. L., Leoni, M. & Scardi, P. (2011). *Z. Kristallogr. Proc.* **1**, 189–194.

[bb17] Gross, D. & Li, M. (2002). *Appl. Phys. Lett.* **80**, 746–748.

[bb18] Guinier, A. (1963). *X-ray Diffraction: In Crystals, Imperfect Crystals, and Amorphous Bodies* San Francisco: W. H. Freeman and Company.

[bb19] Hutchings, M. T., Withers, P. J., Holden, T. M. & Lorentzen, T. (2005). *Introduction to the Characterization of Residual Stress by Neutron Diffraction* Boca Raton: CRC Press, Taylor and Francis Group.

[bb20] Leonardi, A., Beyerlein, K. R., Xu, T., Li, M., Leoni, M. & Scardi, P. (2011). *Z. Kristallogr. Proc.* **1**, 37–42.

[bb22] Leonardi, A., Leoni, M., Li, M. & Scardi, P. (2012). *J. Nanosci. Nanotechnol.* **12**, 8546–8553. 10.1166/jnn.2012.6807 23421242

[bb21] Leonardi, A., Leoni, M. & Scardi, P. (2013*a*). *J. Comput. Mater. Sci.* **67**, 238–242.

[bb24] Leonardi, A., Leoni, M. & Scardi, P. (2013*b*). *Metall. Mater. Trans. A*, **44**, 39–44.

[bb23] Leonardi, A., Leoni, M. & Scardi, P. (2013*c*). *Thin Solid Films* In the press. doi:10.1016/j.tsf.2012.05.037.

[bb25] Leonardi, A., Scardi, P. & Leoni, M. (2012). *Philos. Mag.* **92**, 986–1005.

[bb26] Martinez-Garcia, J., Leoni, M. & Scardi, P. (2009). *Acta Cryst.* A**65**, 109–119. 10.1107/S010876730804186X 19225191

[bb27] Mittemeijer, E. J. & Scardi, P. (2004). *Diffraction Analysis of the Microstructure of Materials* Berlin: Springer-Verlag.

[bb28] Plimpton, S. (1995). *J. Comput. Phys.* **117**, 1–19.

[bb29] Robinson, I. & Harder, R. (2009). *Nat. Mater.* **8**, 291–298. 10.1038/nmat2400 19308088

[bb30] Scardi, P. & Leoni, M. (2001). *Acta Cryst.* A**57**, 604–613. 10.1107/s0108767301008881 11526309

[bb31] Scardi, P. & Leoni, M. (2002). *Acta Cryst.* A**58**, 190–200. 10.1107/s0108767301021298 11832590

[bb32] Sibson, R. (1980). *Scand. J. Stat.* **7**, 14–20.

[bb33] Stokes, A. R. & Wilson, A. J. C. (1944). *Proc. Phys. Soc.* **56**, 174–181.

[bb34] Stukowski, A., Markmann, J., Weissmüller, J. & Albe, K. (2009). *Acta Mater.* **57**, 1648–1654.

[bb35] Suzudo, T. & Kaburaki, H. (2009). *J. Phys. Lett. A*, **373**, 4484–4488.

[bb36] Thomas, O. (2008). *Z. Kristallogr.* **223**, 569–574.

[bb37] Van Houtte, P. (1993). *Mater. Sci. Forum*, **133–136**, 97–110.

[bb38] Van Swygenhoven, H. (2002). *Science*, **296**, 66–67. 10.1126/science.1071040 11935012

[bb39] Van Swygenhoven, H., Farkas, D. & Caro, A. (2000). *Phys. Rev. B*, **62**, 831–838.

[bb40] Warren, B. E. (1955). *Acta Cryst.* **8**, 483–486.

[bb41] Warren, B. E. (1959). *Progr. Met. Phys.* **8**, 147–202.

[bb42] Warren, B. E. (1990). *X-ray Diffraction* New York: Dover.

[bb43] Warren, B. E. & Averbach, B. L. (1950). *J. Appl. Phys.* **21**, 595–599.

[bb44] Warren, B. E. & Averbach, B. L. (1952). *J. Appl. Phys.* **23**, 497–498.

[bb45] Watari, M., McKendry, R. A., Vögtli, M., Aeppli, G., Soh, Y. A., Shi, X., Xiong, G., Huang, X., Harder, R. & Robinson, I. K. (2011). *Nat. Mater.* **10**, 862–866. 10.1038/nmat3124 21946612

[bb46] Wilkens, M. (1969). *Fundamental Aspects of Dislocation Theory*, pp. 1195–1221. Washington, DC: US Government Printing Office.

[bb47] Wilkens, M. (1970). *Phys. Status Solidi A*, **2**, 359–370.

[bb48] Wilson, A. J. C. (1962). *X-ray Optics: the Diffraction of X-rays by Finite and Imperfect Crystals* London: Methuen.

[bb49] Xu, T. & Li, M. (2009). *Philos. Mag.* **89**, 349–374.

